# EDUCATIONAL ATTAINMENT AND LATER-LIFE COGNITIVE FUNCTION IN HIGH- AND MIDDLE-INCOME COUNTRIES

**DOI:** 10.1093/geroni/igae098.1062

**Published:** 2024-12-31

**Authors:** Yuan Zhang, Xuexin Yu, Tsai-Chin Cho, Pengyuan (Kelvin) Zhang, Kenneth Langa, David Weir, Alden Gross, Lindsay Kobayashi

**Affiliations:** Columbia University, New York, New York, United States; University of Michigan, Ann Arbor, Michigan, United States; University of Michigan, Ann Arbor, Michigan, United States; University of Michigan, Okemos, Michigan, United States; University of Michigan, Ann Arbor, Michigan, United States; University of Michigan, Ann Arbor, Michigan, United States; Johns Hopkins Bloomberg School of Public Health, Baltimore, Maryland, United States; University of Michigan, Ann Arbor, Michigan, United States

## Abstract

The rising number of dementia cases worldwide, especially in low- and middle-income countries (LMICs), underscores the need to identify social policies that promote cognitive health of older adults in LMICs and reduce global dementia burden. Many studies have found that educational attainment is associated with better cognitive function. However, these findings are mostly from Western high-income countries where the social and historical contexts of education differ from LMICs. In this research, we aim to evaluate the importance of educational attainment for later-life cognitive function in various social and geographic settings. Using data from the US Health and Retirement Study (HRS) and its sister studies in England, Mexico, China, and India and each study’s respective Harmonized Cognitive Assessment Protocol (HCAP) (n = 14,978, 2016–2019), we compared later-life global cognitive function across countries, examined educational differences in cognitive function within each country, and evaluated the potential impact of improving educational attainment in LMICs on reducing differences in later-life cognitive function between LMICs and high-income countries. In Mexico, China, and India, the general cognitive function score was approximately one standard deviation of HRS-HCAP scores lower compared to the US and England, paralleling patterns of educational attainment across countries. Higher educational attainment was consistently associated with better cognitive function across countries. Differences in educational attainment explained ~50%–90% of observed differences in cognitive function across countries. Our results underscore the importance of education to promote cognitive health. Continual improvement of educational attainment in LMICs may yield significant payoffs in later-life cognitive health.

